# Unraveling the Impact of the Oil Phase on the Physicochemical Stability and Skin Permeability of Melatonin Gel Formulations

**DOI:** 10.3390/gels10090595

**Published:** 2024-09-16

**Authors:** Juan J. Torrado, Brayan J. Anaya, Aytug Kara, Baris Ongoren, Sofía Esteban-Ruiz, Almudena Laguna, Alicia Guillén, Miguel G. Saro, Dolores R. Serrano

**Affiliations:** 1 Pharmaceutics, School of Pharmacy, Complutense University of Madrid, 28040 Madrid, Spain; branaya@ucm.es (B.J.A.); akara@ucm.es (A.K.); bongoren@ucm.es (B.O.); sofiadee@ucm.es (S.E.-R.); almulagu@ucm.es (A.L.); aligui01@ucm.es (A.G.);; 2 Industrial Pharmacy Institute, Complutense University of Madrid, 28040 Madrid, Spain

**Keywords:** melatonin, Pemulen^®^, stability, oxidation, viscosity, skin adhesiveness, QbD, DoE

## Abstract

Melatonin’s antioxidant properties make it a valuable component in anti-aging semisolid topical products. This study explores the role of Pemulen^®^, an acrylic-based viscosifying agent, in stabilizing cream-gel formulations. Remarkably, even at low concentrations (0.4%), Pemulen^®^ successfully produced physicochemical stable topical formulations. In this work, the impact of the ratio of the oily phase—comprising olive oil and isopropyl myristate from 0 to 20%—was investigated to understand the internal microstructure effect on skin permeability, rheological properties, and stability. The formulations exhibited pseudoplastic behavior, with a significant positive correlation (*p*-value < 0.1) between the oily phase ratio, viscosity, spreadability, skin adhesiveness, and permeability. Formulations without the oil phase exhibited greater skin permeability. However, higher oily phase content enhanced viscosity, spreadability, and skin adhesion. Given that melatonin primarily degrades through oxidation, incorporating antioxidant excipients in semisolid formulations is crucial for maintaining its chemical stability. A quality by design (QbD) approach was used to assess the impact of four excipients—(a) DL-α-tocopheryl acetate (0.05%), (b) ascorbic acid (0.1%), (c) ethylene diamine tetraacetic acid (0.1%), and (d) sodium metabisulphite (0.5%)—on melatonin’s stability. Our findings indicate that maintaining the physical stability of the formulation with a 20% oil phase is more critical for protecting melatonin from oxidation than merely adding antioxidant excipients.

## 1. Introduction

During aging, there is a drying process in the skin which can be locally treated by the application of topical formulations [1]. The anti-aging effects of topical formulations can be improved by the addition of antioxidant-active products such as melatonin [2,3,4,5]. However, the physicochemical properties of melatonin make it challenging, considering its poor aqueous stability and low skin permeability [6]. In recent years, the potential of melatonin beyond managing insomnia has been linked to its ability to neutralize the oxidative stress of toxic substances, modulate the inflammatory response, and prevent DNA damage [7,8]. However, much higher concentrations are required to elicit these effects, and thus, there is a need for topical formulations containing much greater concentrations than those currently available in marketed products. The development of physicochemical stable melatonin topical formulations should be guided by the application of quality by design (QbD), targeting good stability balanced with suitable skin permeability to elicit a pharmacological effect.

According to US Pharmacopeia (USP), gels are defined as a semisolid system composed of a dispersion that consists of either small inorganic particles or large organic molecules, which are surrounded and penetrated by a liquid [9]. Structurally, gels are a two-phase system where inorganic particles are dispersed, not dissolved, within the continuous phase, while large organic particles dissolve in the continuous phase, forming randomly coiled, flexible chains [10]. In contrast, creams can be defined as white heterogeneous semisolid topical products generally containing water and oily phases [11]. The incorporation of these two phases agrees with the natural presence of both water and oil secretions on the surface of the skin. Mixtures of both types of water and oil components tend to provide greater hydration and protection of the skin.

To achieve the advantages of both types of formulations, cream-gels have arisen as a hybrid product that combines the characteristics of both gels and creams. It typically has a lightweight, non-greasy texture, offering the hydration and spreadability of creams while providing the cooling, refreshing sensation of a gel. Cream-gels are formulated with polymeric emulsifiers instead of oily ones. However, formulating cream-gels is challenging, as the selected polymeric emulsifier has to balance the oil and water phases to achieve the desired texture while ensuring the product remains stable and effective [12].

In most conventional creams, the viscosity depends on the incorporation of oil components with high melting temperatures, such as cetyl alcohol or other similar excipients [13]. However, these oily creams are not well perceived by consumers and now some of those oil excipients are replaced by water viscosity agents, such as acrylic derivative polymers. Pemulen^®^ is an acrylic derivative viscosity agent that even at small quantities, such as 0.4%, provides gels with high viscosity [14,15,16]. Interestingly, the Pemulen^®^ TR-1 y TR-2 also has surfactant properties which are suitable to obtain stable O/W creams without the need to heat the components to elaborate the products. Cold process elaboration is important to avoid chemical degradation of thermolabile active compounds such as melatonin [17,18]. Another important characteristic of Pemulen^®^ semisolid formulations is that when they come into contact with a saline medium such as skin, their internal structure is changed, and there is a decrease in their viscosity properties. This decrease in viscosity is related to fast component release to the medium and the consequent topical action of the active components of the cream [19,20,21,22]. Currently, it is difficult to investigate the change in viscosity during skin application, which can be defined as the resistance against the movement [23].

The first aim of this work was to study the effect of the oil ratio on the skin permeability, spreadability, and rheological characteristics of melatonin cream-gel formulations using Pemulen^®^ as a thickening agent. The second aim of this work focused on unraveling the impact of the addition of different excipients as potential stabilizers of melatonin. The addition of the following excipients was tested using quality by design: (a) DL-α-tocopheryl acetate (0.05%), (b) ascorbic acid (0.1%), (c) ethylene diamine tetraacetic acid EDTA (0.1%) and (d) sodium metabisulphite (0.5%). Chemical stability was studied by HPLC while physical characteristics were studied through rheological, extensibility, and internal phase particle size characterization.

## 2. Results and Discussion

### 2.1. Effect of Oil Phase on Viscosity and Spreadability

**Figure 1:**
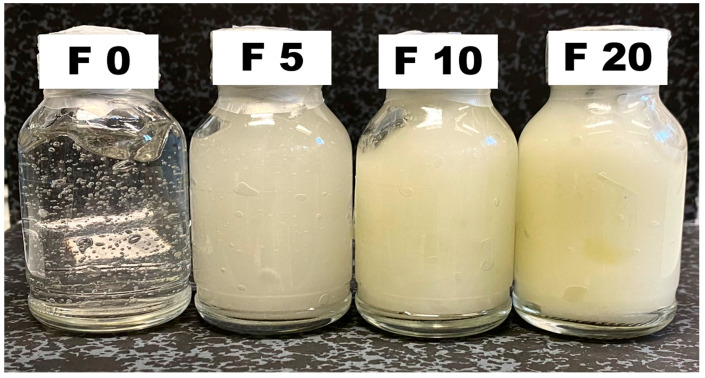
Physical appearance of formulations with different oil ratios. Code: F0 No oily phase, F5—5%, F10—10%, and F20—20% oil phase.

The appearance of semisolid formulations depending on the ratio of the oil phase was investigated (Figure 1). The oil phase was a mixture of transparent isopropyl myristate with yellowish olive oil. The isopropyl myristate was selected as the main component of the oily phase due to its fast-release characteristics [20,24], while olive oil has previously been proved to improve melatonin stability [25] and the transdermal absorption enhancement effect due to the oleic acid content in the olive oil [26]. When no oil phase was added to the formulation (F0), the formulation exhibited a gel-like appearance. However, the addition of the oil phase while stirring leads to emulsification and the formation of cream-gel formulations. The yellow color of the formulation was directly correlated with the final ratio of olive oil in the formulation. The amount of Pemulen^®^ used agrees with other reports ranging from 0.1 to 0.4%. Those percentages are sufficient to stabilize the oil phase. However, Miller and Loffer highlighted that the greater amount of emulsifier does not always correlate to a more stable system, as it is also critical to balance the hydrophobic and hydrophilic counterparts [27]. In our formulation, more than 20% oil phase destabilized the cream-gels.

Traditional ionic or non-ionic surfactants stabilize oil-in-water emulsions primarily by adsorbing to the emulsion interface and forming lamellar liquid crystalline layers, typically requiring surfactant concentrations of 3 to 7%. Achieving good emulsion stability necessitates a careful match between the hydrophilic–lipophilic balance (HLB) of the oil phase and the surfactant. However, cream-gels stabilized with minimal amounts of Pemulen^®^ emulsifiers (<0.5%) were exceptionally stable. In these systems, the oil droplets are surrounded and stabilized by a highly viscous aqueous gel. The long hydrophilic parts of Pemulen^®^ molecules form a micro-gel around each oil droplet, with their hydrophobic segments anchored in the oil phase. As a result, when two oil droplets come close to each other, a physical repulsion is generated by these adsorbed gel layers [28].

**Figure 2:**
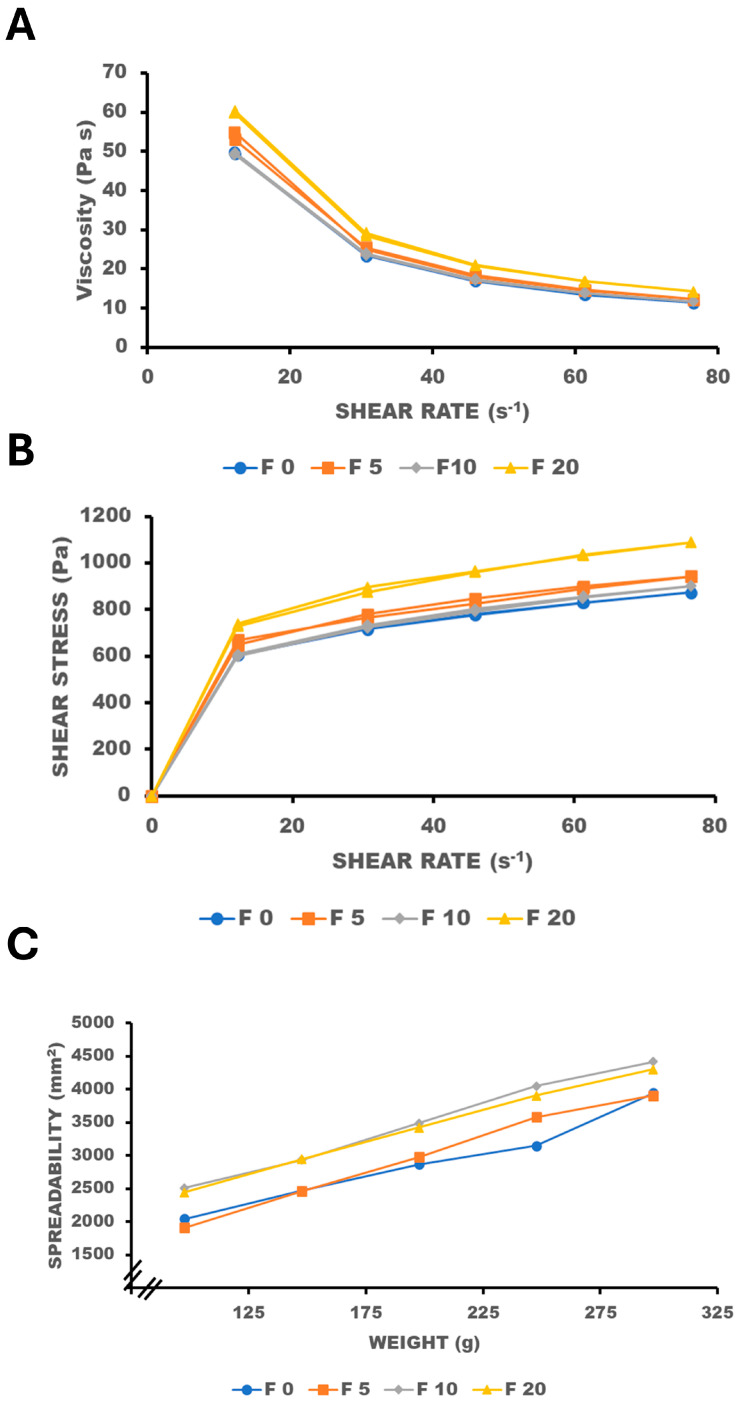
Impact of oil percentage on the flow curves (**A**,**B**) and spreadability (**C**). Code: F0 No oily phase, F5—5%, F10—10%, and F20—20% oil phase.

Figure 2A,B shows the effect of the oily phase ratio on the flow curves of the semisolid formulations. All formulations show a non-Newtonian pseudoplastic behavior similar to those previously reported [20,21,22,23]. Thixotropy values of the formulations were low with a mean value of 11.9 ± 4.2 Pa, similar to low thixotropy values also described by Shahin et al., 2011 [20]. The addition of an oily phase significantly (*p*-value < 0.1) increases the viscosity of the semisolid formulation (Supplementary Material (Figure S1)). Figure 2C shows the spreadability of the formulations depending on the oily phase ratio. Spreadability significantly increased with oily phase ratio (*p*-value < 0.1). In these tested formulations, the oily phase was directly correlated to increasing both the viscosity and the spreadability. Usually, spreadability is inverse to viscosity [29], but in these cream-gel formulations, the lubricant effect of the oily phase was attributed to the greater spreadability. A 20% composition of the oily phase was selected as a suitable formulation for melatonin topical formulations. A 20% oily phase is the upper limit concentration that is also suggested in the technical description of the excipient Pemulen^®^ TR-1 [14]. Melatonin suffers from hydrolysis in aqueous media [30]. We hypothesized that the incorporation of melatonin within the oil fraction of the cream-gel could enhance its physicochemical stability. However, at this ratio of oily phase, the physical stability of the formulations could be compromised, making the addition of preservatives necessary.

### 2.2. Effect of Oil Phase on Skin Permeability

**Figure 3:**
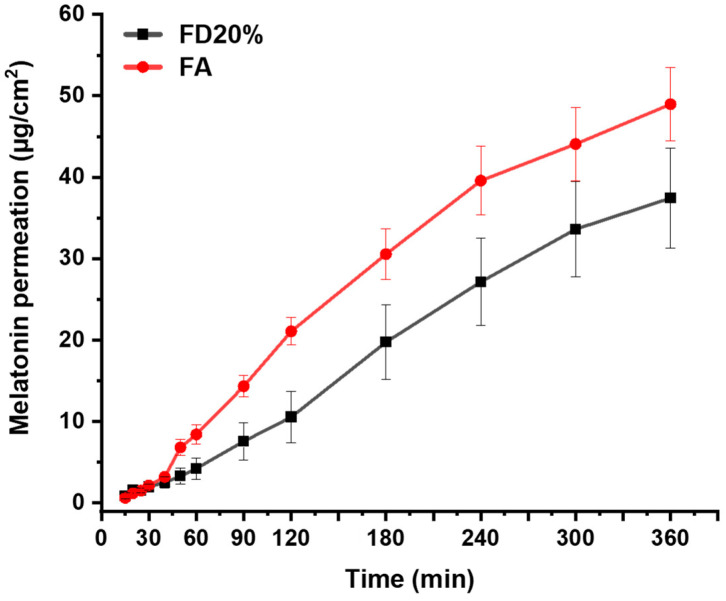
Skin permeability of F0 (-●-) and F20 (-■-) cream-gel formulations.

Cream-gel formulations with the lowest (F0) and the highest (F20) oil phase ratio were compared in terms of skin permeability. It is worth noting that F0 enhanced melatonin skin permeability and significantly reduced the lag time which can be attributed to the lower viscosity (Table 1 and Figure 3).

In both cases, the permeability flux rates for melatonin across the skin were significantly higher, ranging between 1.13 and 1.52 µg/cm^2^/min, than the values reported for melatonin cream formulations based on Cera Lanette^®^ N (0.065 µg/cm^2^/min) [6]. This indicates the impact of the organization of the internal phase in the permeability across the pig skin, particularly the stratum corneum. Pemulen^®^ polymeric emulsifiers are innovative oil-in-water (o/w) emulsifiers that are primarily composed of high molecular weight polyacrylic acid polymers. As novel primary emulsifiers, they feature a unique chemical structure with a small lipophilic segment alongside a large hydrophilic segment. This structure enables the copolymers to effectively act as primary emulsifiers in oil-in-water emulsions. The lipophilic segment adsorbs at the oil-water interface, while the hydrophilic segment swells in the water, forming a gel network around the oil droplets [31]. However, in the F0 with 0% oily phase, melatonin is readily available to go across the stratum corneum compared with the F20, which explains the lower T_lag_.

### 2.3. Effect of Oil Phase on Skin Adhesion

**Figure 4:**
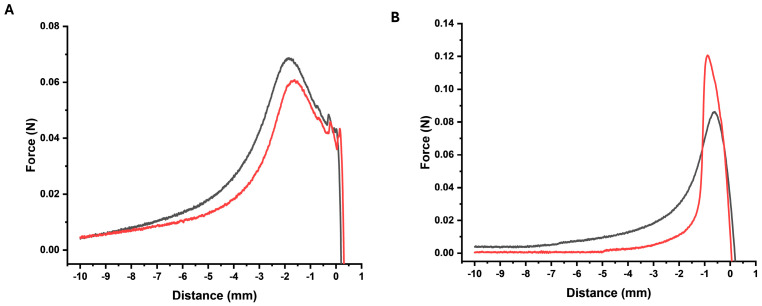
Skin adhesiveness for F0 (**A**) and F20 (**B**) melatonin cream-gel formulations (*n* = 2 in red and black). The peak represents the maximum force required to detach the probe from the gel.

The effect on skin adhesiveness showed an opposite behavior between the F0 and the F20. The latter showed a 2-fold higher adhesive force to the skin compared with F0, and hence, this can be correlated with the likelihood of remaining for longer periods on the skin (Figure 4).

To achieve adhesion, a polymer must possess at least one of the following characteristics: (i) an adequate number of hydrogen bond-forming groups, such as hydroxyl or carboxyl groups; (ii) an anionic surface charge; (iii) a high molecular weight; (iv) significant chain flexibility; or (v) surface tension properties that promote spreading into the adhesive layer [32]. These characteristics are met by the two different polymers of Pemulen^®^; TR1 with the higher polymerization-degree polymer used in this work, and TR2 which exhibits a lower degree of polymerization [33]. Cream-gels using TR2 have lower elasticity and are more capable of forming bonds with the surfaces than TR1, which has higher elasticity making its spreadability easy. Above 0.2% of TR1, the adhesiveness decreases with the amount of the TR1 polymer but increases when using TR2 [34]. In this work, we balanced the spreadability and the skin adhesiveness by combining 0.4% Pemulen TR1 with 20% oil phase to ensure a prolonged effect with a better skin feeling.

### 2.4. Stability of Melatonin Topical Formulations

Based on the previous results, even though F0 showed higher skin permeability, F20 was selected as a better choice for topical delivery of melatonin considering the poor stability in aqueous media and higher tendency to degrade of F0, while within the oily droplets of F20 is expected to improve its stability. Based on this consideration, as well as the better adhesiveness of the skin, a quality-by-design approach was applied to the F20 to improve its physicochemical stability by incorporating a range of different stabilizers.

**Figure 5:**
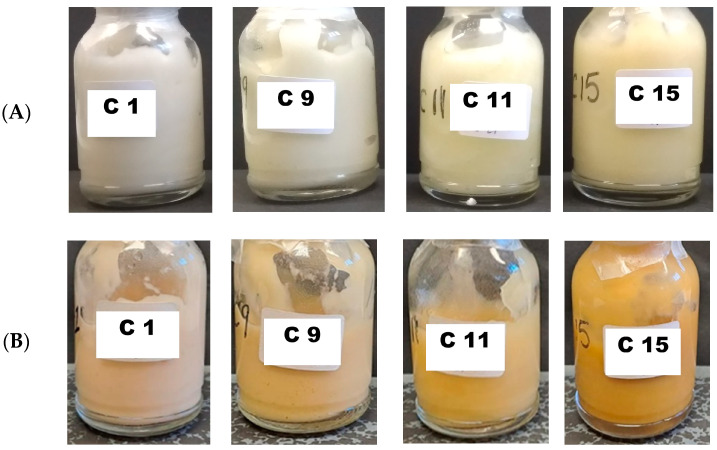
Physical appearance of the most stable melatonin cream-gels of melatonin from the QbD. The top panel illustrates the initial appearance one day after preparation (Panel **A**). The down panel represents the appearance after 18 months of storage (Panel **B**). The composition of the formulations C1, C9, C11 and C15 is described in the methodology section in Table 5.

Only four of the sixteen melatonin creams with 20% oily phase remained physically stable after one year of storage. Figure 5 shows the physical appearance of the stable melatonin creams initially (Panel A), and after 18 months of storage (Panel B). In most of the cream-gel formulations, the initial appearance was white, but for those with vitamin E (variable A in the experimental formulation design), the color was pale green depending on the presence or not of vitamin E. All tested formulations showed a change in physical appearance after 12 months of storage related to melatonin oxidation. However, hydrolysis and oxidation were related to both the chemical composition of the cream and its physical instability.

**Figure 6:**
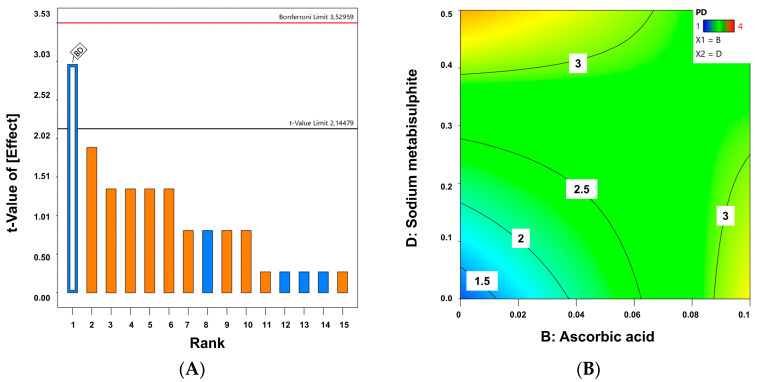
Pareto Chart (**A**) and 2D contour plot (**B**) on the physical stability of melatonin. Factor B (ascorbic acid) and D (sodium metabisulphite) are expressed in percentage (%). Orange bars represent a positive effect of the factor on the response while blue bars represent a negative effect.

Figure 6 shows the effect of the different variables on the physical stability of the melatonin formulations. The Pareto Chart (Figure 6A) clearly indicates that the combination of the BD factors (ascorbic acid and sodium metabisulphite) are those playing a critical role (*p*-values: 0.0041). The lower the percentage of sodium metabisulphite and ascorbic acid, the better the physical appearance (R^2^ = 0.672) (Figure 6B).

**Figure 7:**
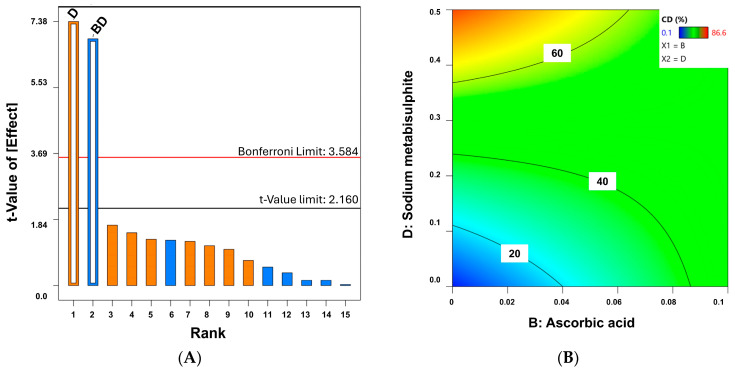
Pareto Chart (**A**) and 2D contour plot (**B**) on the chemical stability of melatonin (expressed as melatonin degraded in percentage). Factor B (ascorbic acid) and D (sodium metabisulphite) are expressed in percentage (%). Orange bars represent a positive effect of the factor on the response while blue bars represent a negative effect.

The effect of the four independent variables on the chemical degradation of melatonin is depicted in Figure 7. The Pareto Chart (Figure 7A) indicates that the percentage of D (sodium metabisulphite) has the most significant impact on the chemical stability of melatonin, followed by the combination with ascorbic acid (*p*-values < 0.0001). Similar to the physical degradation, the lower the ascorbic acid and the sodium metabisulphite, the lower the degradation, and hence the greater the chemical stability of melatonin (Figure 7B). A better correlation was obtained for the chemical degradation than the physical appearance (R^2^ = 0.895). Antioxidants can also act as pro-oxidants under certain circumstances [35]. This highlights the importance of selecting suitable antioxidants in adequate concentrations in topical formulations because, due to their instability, keeping the activities of the antioxidants constant during the shelf life of the formulation can be problematic [36].

**Figure 8:**
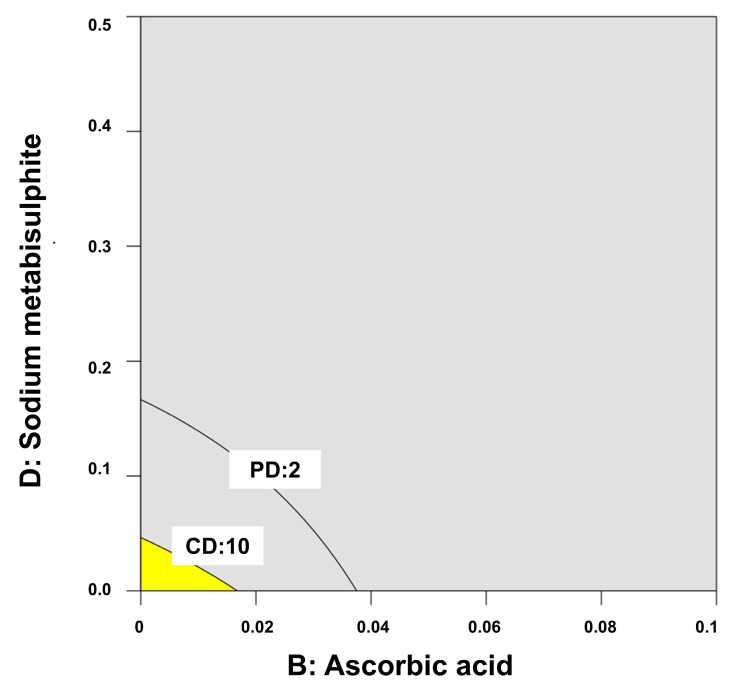
Overlay plot indicating in yellow the optimal formulation design of space. Factor B (ascorbic acid) and D (sodium metabisulphite) are expressed in percentage (%). Key: PD is Physical Degradation and CD is Chemical Degradation (expressed as the percentage of degraded melatonin).

The optimal design of space was delimited by the percentage of sodium metabisulphite and ascorbic acid (Figure 8). The content of EDTA and DL-α-tocopheryl acetate did not play a major role in the physicochemical stability of the melatonin, with C1, C9, C11 and C15 being those with the best performance.

**Figure 9:**
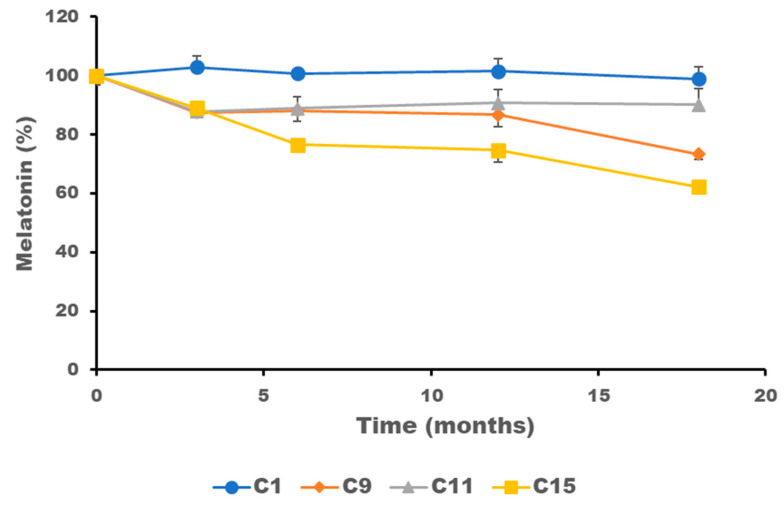
Mean results and standard deviation (*n* = 3) of the chemical degradation of melatonin (expressed as % of initial content) during 18-month storage. The composition of the formulations C1, C9, C11 and C15 is described in the methodology section in Table 5.

C1, C9, C11 and C15 formulations showed the best physical stability with minimal color change and no phase separation observed. The melatonin degradation profile of these formulations is illustrated in Figure 9, with Formulation C1 being the most chemically stable. Interestingly, the simpler the formulation, regardless of the addition of excipients, the more physical and chemical stability.

**Figure 10:**
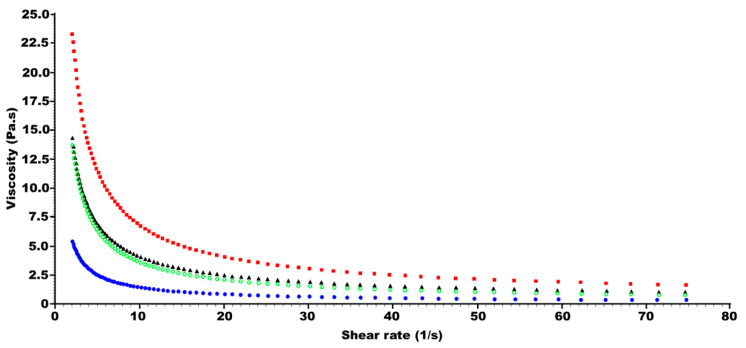
Rheograms of melatonin semisolid formulations C1 (red), C9 (blue), C11 (black), and C15 (green) after 18 months of storage at 22 °C.

Rheograms of those four formulations after 18 months of storage are shown in Figure 10. The physical characteristics of the four more stable formulations after 18 months of storage are reported in Table 2. Viscosity results are significantly (*p*-value < 0.01) correlated to the mean volume size of the internal phase and the standard deviation. The lower the size of the internal phase and the lower the standard deviation (which results in more homogenous particles with better packing), the higher the viscosity. Formulation C1 exhibited the lowest particle size and standard deviation resulting in the greater viscosity, which led to the best physical and chemical stability.

The smallest and more homogenous particle size of the internal phase can increase the number of particle–particle interactions, which translates into higher viscosity. Nevertheless, this significantly higher viscosity is only observed at low shear rates attributed to the weak nature of these particle–particle interactions (Figure 10).

## 3. Conclusions

Formulation C1 showed the best physical and chemical stability correlation, even at high percentages of oil phase (up to 20%), in combination with Pemulen^®^ TR-1. The main advantage of combining Pemulen^®^ with a 20% oil phase is that a minimum amount of additional surfactant and stabilizer excipients are required to ensure optimal physical rheological characteristics in terms of viscosity and spreadability in being suitable for cosmetic application. This suggests that Pemulen^®^ melatonin cream-gel formulations with 20% oil phase could be a promising option for topical delivery with enhanced physicochemical stability long-term.

## 4. Materials and Methods

### 4.1. Materials

Melatonin (Ph. Eur. Grade) was purchased from Fagrón Ibérica SAU (Madrid, Spain). All excipients were of Pharmacopoeia grade. Pemulen^®^ TR-1 was supplied by Lubrizol (Madrid, Spain). Propylparaben, glycerine, olive oil and DL-α-tocopheryl acetate were purchased from Fagrón Ibérica SAU (Madrid, Spain). Isopropyl myristate, sodium EDTA, triethanolamine, ethanol 96° and sodium metabisulphite were supplied by Panreac AppliChem (Barcelona, Spain). Methylparaben was purchased from Acofarma (Madrid, Spain), ascorbic acid was supplied by Guinama (Valencia, Spain) and purified water was obtained through Elix-3^®^ (Merck Millipore, Burlington, MA, USA). All other chemicals were of ACS reagent grade or above from Panreac AppliChem (Barcelona, Spain), and solvents were of HPLC grade (Scharlau, Madrid, Spain) and were used as supplied.

### 4.2. Preparation of Topical Melatonin Formulations with Different Oily Phase Ratio

A prescreening formulation development was performed with a range of excipients to investigate the effect of the amount of oily phase on the rheological and spreadability characteristics of topical melatonin formulations. Table 3 shows the composition of the prescreening formulations. All formulations contained 0.1% melatonin and 0.4% Pemulen^®^ TR-1 as the emulsifying agent. Methylparaben and propylparaben were included as the preservatives dissolved in ethanol. Different ratios of isopropyl myristate and olive oil were incorporated, the latter ranging from 0 (F0 formulation) to 20% (F20 formulation).

The topical formulations were prepared in batches of 600 mL with a conventional magnetic stirrer (Ika, Barcelona, Spain). First, methyl and propyl parabens were dissolved in ethanol. Glycerine and melatonin were then added and mixed with approximately three-quarters of the deionized water. Pemulen^®^ was incorporated and left to swell for 12 h. The oil phase (olive oil and isopropyl myristate) was prepared separately and then mixed with the water phase under constant stirring to form an O/W emulsion. Finally, triethanolamine was added to adjust the pH to 5.6 ± 0.3.

### 4.3. Rheology of Topical Melatonin Formulations with Different Oily Phase Ratio

Rheological characteristics were studied with a Brookfield DV II viscometer (Brookfield Engineering Laboratories, MA, USA) with a spindle 5 RV at 22 ± 1 °C. The speed of the spindle was changed from 0 to 25 rpm and then back to 0 rpm. Shear rates were from 0 to 76 s^−1^ increasing and decreasing sequentially to obtain a rheogram of each formulation. Viscosity (η) and torque values are directly provided by the equipment, while shear stress (σ) and shear rates (γ) are estimated based on the Herschel-Bulkley Equation (1) [21]:

σ = σy + K × γn (1)

where K is a viscosity coefficient and n is a pseudoplasticity index. Thixotropy (Pa) was evaluated as the difference in the area between the ascending and descending curves between viscosity and shear rate [29].

For the spreadability test, the surface area (mm^2^) of a semisolid formulation varies directly according to the weight applied over the formulation [37]. Approximately 1 g of the formulation was deposited on the surface of a glass plate, then another plate was placed over the formulation, and finally, different weights of 50, 100, 150 and 200 g were placed on top. The surface (mm^2^) of the formulation was correlated with the spreadability performance.

### 4.4. In Vitro Skin Permeation

The skin permeation with the lowest (F0) and the highest (F20) percentage of oil phase was assayed. Diffusion studies were performed using vertical diffusion Franz cells (Soham Scientific, Loughborough, UK) as previously described [38,39]. In vitro skin permeation experiments were performed using pig ear skin (0.85 mm ± 0.12 mm) which was mounted between the donor and receptor chambers of Franz diffusion cells (Soham Scientific, Soham, UK) with an effective diffusion area of 1.76 cm^2^ [40]. Pig ears were obtained from a local pork slaughterhouse (Madrid, Spain). A stirring bar (3 × 5 mm was added to each Franz cell’s receptor compartment, which was filled with 12 mL of fresh PBS at pH 7.4 and maintained at 32 °C ± 0.5 °C with continuous stirring at 350 rpm. Accurately weighed formulations (1 g) were loaded into the donor chambers and spread as a thin layer over the pig ear skin. At predetermined intervals (15, 20, 25, 30, 40, 60, 90, 120, 180, 240, 300, and 360 min), 1 mL samples were withdrawn from the receptor chambers for HPLC analysis without dilution. The withdrawn volumes were immediately replaced with fresh PBS to maintain sink conditions. The cumulative amounts of melatonin permeated through the pig ear skin were plotted as a function of time [38]. Formulations were tested in triplicate. Regression analysis calculated the slopes and intercepts of the linear portion of each graph. The steady-state flux was calculated for each formulation using Equation (2).

(2) $$Jss=\frac{dC}{dX}\times A$$

where *Jss* is the steady-state flux (μg/cm^2^/h), *dC*/*dX* is the amount of melatonin permeating the membrane over time (μg/h), and *A* is the surface area of contact of the formulation [41]. The permeability coefficient (P) was calculated by using Equation (3):

(3) $$P=\frac{Jss}{cd}$$

where *cd* is the amount of drug applied in the donor compartment (1 g of gel formulations equivalent to 0.001 g of melatonin). The diffusion coefficient was calculated by using the following Equation (4):

(4) $$Jss=\frac{D\times k}{h}cd$$

where *h* is the thickness of the skin (0.85 cm) [42,43].

### 4.5. Skin Adhesion

The in vitro adhesiveness of the F0 and F20 melatonin formulations was evaluated in duplicate using a Texture Analyzer TA.XT Plus C (Stable Micro Systems Ltd., Surrey, UK). To measure adhesive strength, the force required to detach the probe from the skin with the formulation applied on the skin was determined. A 3 × 3 cm^2^ pig skin was securely mounted on the base of the texture analyzer. A 0.5 g formulation (F0 or F20) was placed on the surface of the skin. A cylindrical probe with a diameter of 0.5 inches (p/0.5) was then driven into the gel at a constant speed of 0.5 mm/s. Upon contact with the skin, a 49 mN force was applied for 5 s, after which the probe was detached at a post-test speed of 10 mm/s. Data was collected at a rate of 200 points per second (PPS). The maximum force recorded to detach the probe from the skin was used to quantify the melatonin formulation’s adhesion to the pig skin, and was calculated using Exponent software (version 8.0.14.0). The results were plotted using Origin 2021 (OriginLab Corporation, Northampton, MA, USA) [44,45].

### 4.6. Quality by Design for Optimization of Physicochemical Stability of Melatonin Topical Formulations

F20 formulation was selected from the above composition, and a design of experiments was performed in detail to find the optimal formulation for the best physicochemical stability. The quality target product profile (QTPP) was focused on a formulation with optimal physical appearance after at least 12 months of storage with no melatonin chemical degradation [46]. In this QbD, several critical material attributes (CMAs) were identified due to their antioxidant properties to prevent melatonin oxidation. The following four excipients were studied as independent variables: (a) DL-α-tocopheryl acetate (0.05%), (b) Ascorbic acid (0.1%), (c) EDTA (0.1%), and (d) Sodium metabisulphite (0.5%). A regular two-level DoE was applied (2^4^). Table 4 shows the identified QTPP and CMAs, while Table 5 shows the matrix for the tested formulations correlated with the stability of melatonin.

The topical formulations were elaborated as described in Section 4.2. Depending on the DoE matrix, DL-α-tocopheryl acetate, ascorbic acid, EDTA, and sodium metabisulphite were either included or not. Transparent 70 mL glass vials were used for the primary packaging and stored protected from light, kept at 22 ± 1 °C, and assayed at 3, 6, 12, and 18 months for physical and chemical melatonin stability.

Mathematical modeling was performed using multiple linear regression analysis (MLRA). In constructing the polynomial equations, only statistically significant coefficients (*p* < 0.05) were included. The model’s performance was assessed by examining the *p*-value, and the coefficient of determination. To explore the relationships between various factors and responses, response surface analysis was conducted using 2D contour plots and Pareto Charts [47]. An overlay plot showing the optimal design space in yellow color was calculated considering the physical degradation to be within the range of 1–2 and the chemical degradation no higher than 10%.

### 4.7. Physical Characterization

The color and visual appearance of the semisolid formulations were evaluated. Physical instability related to appearance and color change was quantified according to the following criteria: 0 for those formulations with the same appearance as the initial one, 1 for those formulations with a slightly darker appearance, 2 for formulations that exhibited a darker color and with partial phase separation, 3 for those formulations that exhibited a brownish color and clear phase separation observed, and 4 for those formulations that exhibited a brownish color and complete phase separation occurred.

Final rheological characteristics during stability studies were performed in triplicate using an AR2000 Rheometer (TA Instruments, New Castle, DE, USA) and a 4 cm flat plate geometry. The rheology was tested according to the evolution of shear stress versus shear rate. The rheometer was configured to increase the shear rate by 0.33 Pa/s up to 75 s^−1^. The collected data was analyzed using TA Universal Analysis software (TA Instruments, New Castle, DE, USA) [6].

Internal phase size was measured after dilution with deionized water (1/1000, *v*/*v*) by laser light diffraction (Zetatrac 3500 Ultra, Microtrac Inc., Montgomeryville, PA, USA) to determine the Mean Volume size (MV) and SD.

### 4.8. Chemical Characterization by HPLC Melatonin Quantification

The formulation (0.1 g) was dispersed in a 2% sodium chloride solution (10 mL) and then diluted with a mixture of HPLC methanol and deionized water (25:75 *v*/*v*). The mixture was homogenized, filtered, and assayed by HPLC according to a validated method described by USP 38 [9]. The HPLC was on Jasco modular equipment. The stationary phase was a C18 column (Waters Spherisorb^®^ S10 ODS1 (Madrid, Spain), 4.6 × 200 mm^2^). The mobile phase was a mixture of 75:25 (buffer: acetonitrile, *v*/*v*). The buffer was prepared by dissolving 0.5 g KH_2_PO_4_ in 1 L of purified water with orthophosphoric acid to adjust the pH to 3.5. The isocratic flow rate was 1.5 mL/min. The retention time for the melatonin was approximately 7.7 min. The injection volume was 10 µL and the detection wavelength was 222 nm [6]. The HPLC was based on the USP. Linearity was studied between 10 and 125% of the theoretical melatonin concentration. The correlation coefficient was 0.99. Repeatability and reproducibility were 1.6 and 4.7%, respectively. Accuracy and intermediate precision on three different days were 102.9 and 4.5%, respectively. Limits of detection (LOD) and quantification (LOQ) were estimated according to the ratio signal noise and were of 0.01 and 0.04 µg/mL, respectively. Examples of chromatograms are provided in the Supplementary Material, including a chromatogram corresponding to a blank sample of the melatonin (Figure S1), chromatograms of the melatonin (with a retention time 7.7 min) without hydrogen peroxide exposition for 12 days (Figure S2) and after hydrogen peroxide exposition showing an oxidation product of the melatonin at a retention time of 5.4 min (Figure S3), a chromatogram of a reference sample of melatonin at 5 µg/mL concentration (Figure S4), and a chromatogram of cream-gel formulation C1 after 18 months of storage, evidencing that no interferences related to the excipients were observed in the stability assay of the different melatonin cream-gel formulations (Figure S5).

### 4.9. Statistics

Skin permeability and chemical stability experiments were performed in triplicate while the skin adhesion test was carried out in duplicate. Anova (for chemical stability) and Student’s two-tailed paired *t*-test (skin permeability and adhesion) were performed with Excel (Office 365, Microsoft).

## Figures and Tables

**Table 1:** Comparison of skin permeation of cream-gel formulations (F20 and F0) with pig ear skin.

Membrane	Jss (µg/cm^2^/min)	Lag time (min)	P (cm/h) × 10^2^	D (cm^2^/h) × 10^3^
F20	1.13 ± 0.38	16.33 ± 8.52	6.80 ± 2.32	0.57 ± 0.16
F0	1.52 ± 0.29	2.31 ± 0.54	9.12 ± 1.77	7.20 ± 1.59

**Table 2:** Physical characteristics of the four most stable melatonin creams after 18 months of storage. The composition of the formulations is described in Table 5 Key code: MV is the mean volume size of the internal phase and SD is the standard deviation of the mean volume size.

Formulation	Viscosity (Pa·s)	MV (µm)
C1	1.8 ± 0.4	21.2 ± 14.4
C9	1.1 ± 0.3	28.3 ± 21.0
C11	0.8 ± 0.2	43.8 ± 22.7
C15	0.3 ± 0.1	34.0 ± 16.8

**Table 3:** Composition of the prescreening melatonin semisolid formulations with different oily phase content. Quantities are expressed in percentages.

Component	F0	F5	F10	F20
Melatonin	0.1	0.1	0.1	0.1
Methylparaben	0.16	0.16	0.16	0.16
Propylparaben	0.04	0.04	0.04	0.04
Ethanol 96	1.0	1.0	1.0	1.0
Glycerine	3	3	3	3
Pemulen^®^ TR-1	0.4	0.4	0.4	0.4
Olive oil	0	1.25	2.5	5
Isopropyl myristate	0	3.75	7.5	15
Triethanolamine	pH 5.6 ± 0.3	pH 5.6 ± 0.3	pH 5.6 ± 0.3	pH 5.6 ± 0.3
Deionized water	Up to 100 mL	Up to 100 mL	Up to 100 mL	Up to 100 mL

**Table 4:** QTPP and CMAs identified for the melatonin topical formulations.

QTPP	CMAs
Physical appearance after at least 12 months of storage (no color change or phase separation)	DL-α-tocopheryl acetate (absence or 0.05%)
	Ascorbic acid (absence or 0.1%)
Low chemical degradation (<10%)	EDTA (absence or 0.1%)
	Sodium metabisulphite (absence or 0.5%)

**Table 5:** Composition of melatonin cream-gel topical formulations, physical and chemical stability after one year. Code of variables: (a) DL-α-tocopheryl acetate (0.05%), (b) Ascorbic acid (0.1%), (c) EDTA (0.1%) and (d) Sodium metabisulphite (0.5%). P.D. is Physical Degradation and C.D. is Chemical Degradation (expressed as the percentage of degraded melatonin). Key: the addition (+) or not (-) of the different variables.

Code Formulation	a	b	c	d	P.D.	C.D.
C1	-	-	-	-	1	0.1
C2	-	-	-	+	4	81.1
C3	-	-	+	-	2	0.5
C4	-	-	+	+	3	72.7
C5	-	+	-	-	4	39.4
C6	-	+	-	+	2	44.9
C7	-	+	+	-	3	57.2
C8	-	+	+	+	2	62.5
C9	+	-	-	-	1	1.4
C10	+	-	-	+	3	86.6
C11	+	-	+	-	1	9.1
C12	+	-	+	+	4	81.6
C13	+	+	-	-	4	50.5
C14	+	+	-	+	3	24.3
C15	+	+	+	-	2	36.2
C16	+	+	+	+	4	62.2

## Data Availability

The data presented in this study are available on request from the corresponding author.

## Supplementary Materials

The following supporting information can be downloaded at: https://www.mdpi.com/article/10.3390/gels10090595/s1, Figure S1. Chromatogram of a blank sample of the melatonin assay method; Figure S2. Chromatogram of melatonin (retention time 7.7 min) after 12 days of exposition in aqueous medium without hydrogen peroxide; Figure S3. Chromatogram of melatonin (RT 7.7 min) after 12 days of exposition in aqueous medium with hydrogen peroxide. Degradation product by oxidation with a RT of 5.4 min; Figure S4. Chromatogram of reference melatonin (RT 8.3 min) at 5 µg/mL; Figure S5. Chromatogram of cream-gel formulation C1. Retention time of melatonin is 8.8 min and retention time of methylparaben is 11.4 min.
