# Supplementary material for: Unraveling the Impact of the Oil Phase on the Physicochemical Stability and Skin Permeability of Melatonin Gel Formulations
Source: Gels. 2024 Sep 16;10(9):595. doi: 10.3390/gels10090595 (PMC11431383; doi:10.3390/gels10090595)
Supplement: Supplementary file 1 [file gels-10-00595-s001.zip › gels-3177957-supplementary.pdf]

Supplementary file of Gels

Article

Unraveling the impact of the oil phase on the physicochemical stability and skin permeability of melatonin gel formulations

Juan J. Torrado[1#], Brayan J. Anaya[1#], Aytug Kara[1], Baris Ongoren[1], Sofía Esteban-Ruíz[1], Almudena Laguna[1], Alicia Guillén[1], Miguel G. Saro[1] and Dolores R. Serrano[1,2,*]

[1] Pharmaceutics, School of Pharmacy, Complutense University of Madrid, Spain; torrado1@ucm.es (JJT), akara@ucm.es (AK); bongoren@ucm.es (BO), almulagu@ucm.es (AL), aligui01@ucm.es (AG), misaro@ucm.es (MS), drserran@ucm.es (DRS)

[2] Industrial Pharmacy Institute, Complutense University of Madrid, Spain; drserran@ucm.es (DRS)

* Correspondence: torrado1@ucm.es (JJT); drserran@ucm.es (DRS)

\# First-coauthorship shared

HPLC assay of melatonin in cream-gel formulations

The method was developed based on the HPLC method described on the USP 38 [9].

Examples of chromatograms are provided below. Figures S1 shows a Chromatogram corresponding to a blank sample of the melatonin HPLC method. Figures S2 and S3 show chromatograms of melatonin (retention time 7.7 minutes) without and with hydrogen peroxide exposition for 12 days. Figure S3 shows an oxidation product of melatonin at a retention time of 5.4 minutes.

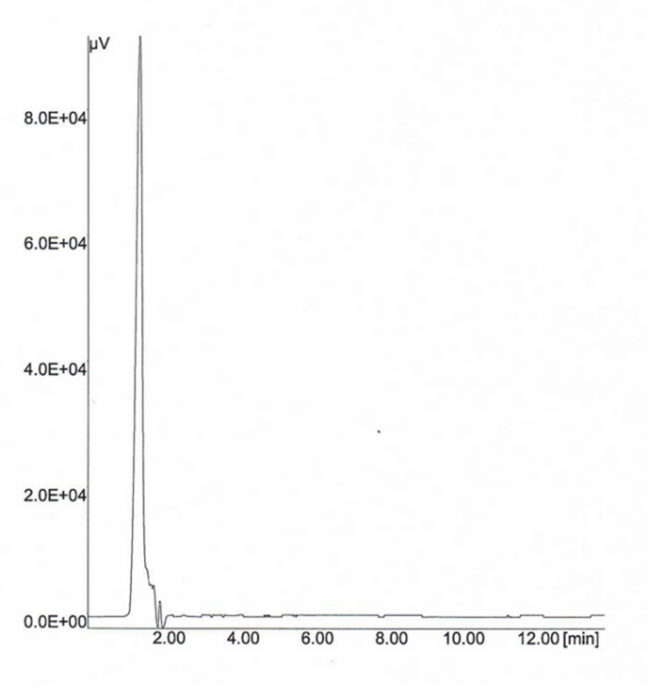

Figure S1. Chromatogram of a blank sample of the melatonin assay method.

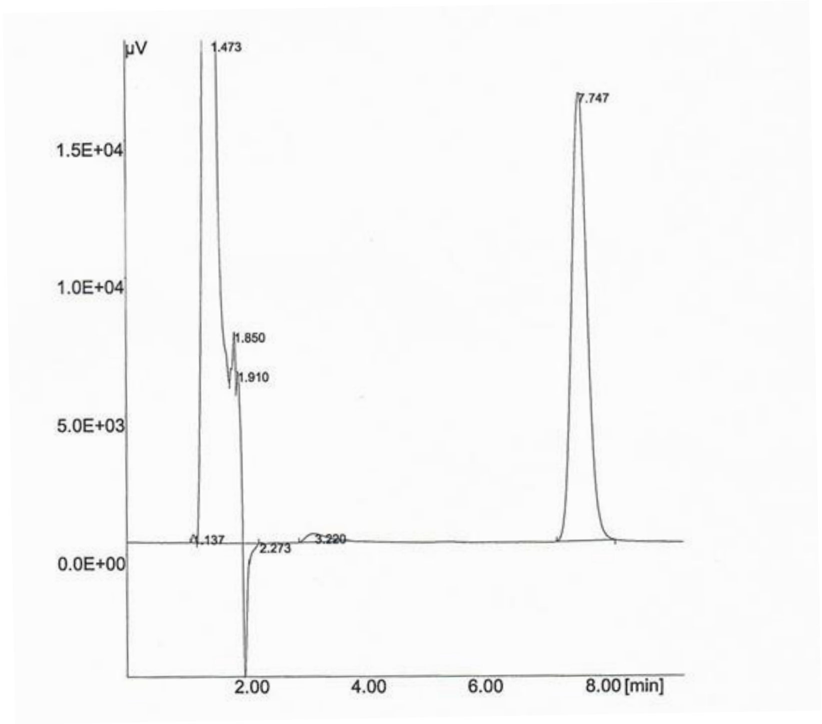

Figure S2. Chromatogram of melatonin (retention time 7.7 min) after 12 days of exposition in aqueous medium without hydrogen peroxide.

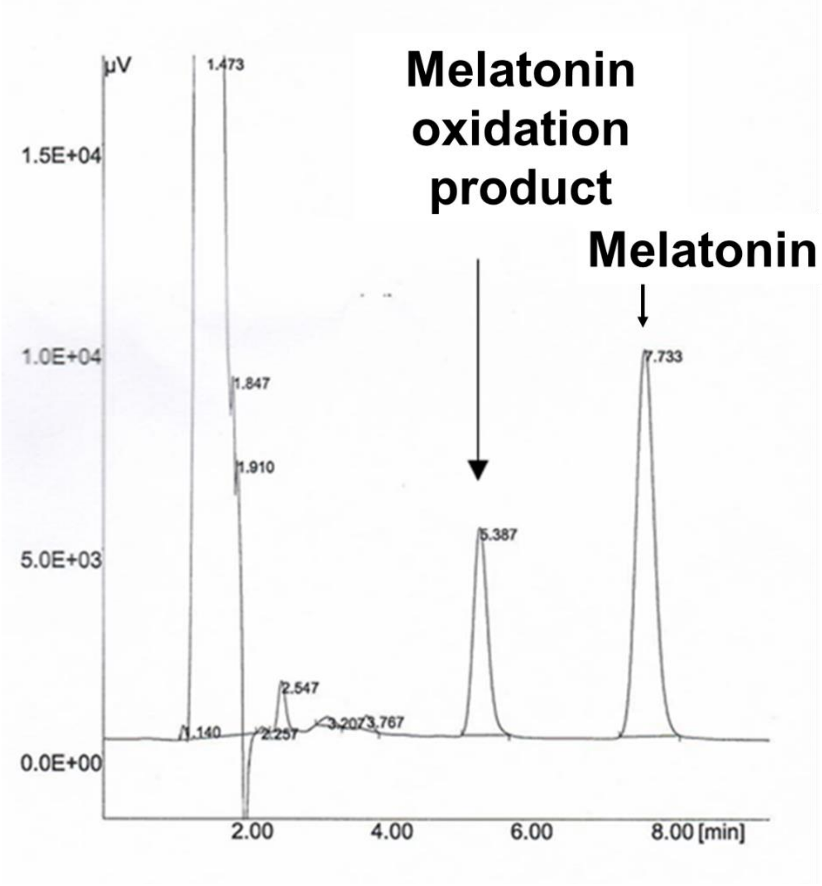

Figure S3. Chromatogram of melatonin (RT 7.7 min) after 12 days of exposition in aqueous medium with hydrogen peroxide. Degradation product by oxidation with a RT of 5.4 minutes.

Figure S4 shows a chromatogram of a reference sample of melatonin at 5 µg/mL concentration. Figure S5 shows a chromatogram of cream-gel formulation C1 after 18 months of storage. No interferences related to the excipients are observed in the assay of the different melatonin cream-gel formulations.

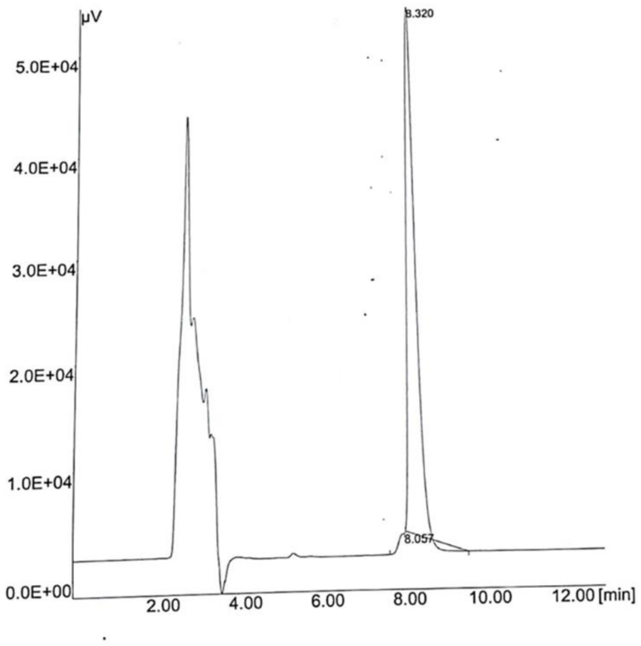

Figure S4. Chromatogram of reference melatonin (RT 8.3 min) at 5 µg/mL.

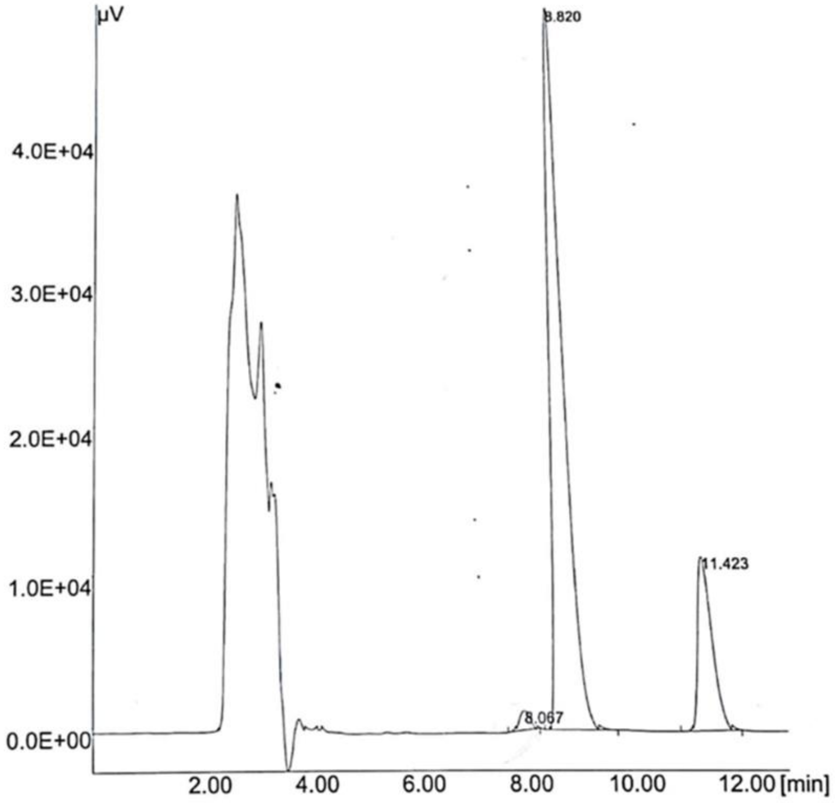

Figure S5. Chromatogram of cream-gel formulation C1. Retention time of melatonin is 8.8 minutes and retention time of methylparaben is 11.4 minutes.
